# The Power of Extracellular Vesicles in Myeloproliferative Neoplasms: “Crafting” a Microenvironment That Matters

**DOI:** 10.3390/cells10092316

**Published:** 2021-09-04

**Authors:** Lucia Catani, Michele Cavo, Francesca Palandri

**Affiliations:** 1 IRCCS Azienda Ospedaliero-Universitaria di Bologna, Department of Experimental, Diagnostic and Specialty Medicine, School of Medicine, Institute of Hematology “Seràgnoli”, University of Bologna, 40138 Bologna, Italy; michele.cavo@unibo.it; 2 IRCCS Azienda Ospedaliero-Universitaria di Bologna, Institute of Hematology “Seràgnoli”, 40138 Bologna, Italy; francesca.palandri@unibo.it

**Keywords:** myeloproliferative neoplasms, extracellular vesicles, essential thrombocythemia, polycythemia vera, myelofibrosis, biomarker, thrombosis, inflammatory microenvironment

## Abstract

Myeloproliferative Neoplasms (MPN) are acquired clonal disorders of the hematopoietic stem cells and include Essential Thrombocythemia, Polycythemia Vera and Myelofibrosis. MPN are characterized by mutations in three driver genes (*JAK2, CALR* and *MPL*) and by a state of chronic inflammation. Notably, MPN patients experience increased risk of thrombosis, disease progression, second neoplasia and evolution to acute leukemia. Extracellular vesicles (EVs) are a heterogeneous population of microparticles with a role in cell-cell communication. The EV-mediated cross-talk occurs via the trafficking of bioactive molecules such as nucleic acids, proteins, metabolites and lipids. Growing interest is focused on EVs and their potential impact on the regulation of blood cancers. Overall, EVs have been suggested to orchestrate the complex interplay between tumor cells and the microenvironment with a pivotal role in “education” and “crafting” of the microenvironment by regulating angiogenesis, coagulation, immune escape and drug resistance of tumors. This review is focused on the role of EVs in MPN. Specifically, we will provide an overview of recent findings on the involvement of EVs in MPN pathogenesis and discuss opportunities for their potential application as diagnostic and prognostic biomarkers.

## 1. Introduction

### 1.1. Myeloproliferative Neoplasms

Myeloproliferative Neoplasms (MPN) are acquired clonal disorders of the hematopoietic stem cells (HSC) and include Essential Thrombocythemia (ET), Polycythemia Vera (PV) and Myelofibrosis (MF). Despite common biological characteristics, MPN patients display disease phenotype heterogeneity due to acquired factors that affect the HSC, and also as a result of heterogeneity in the HSC in which MPN-initiating mutations arise. MF may arise de novo (Primary Myelofibrosis, PMF) or after Essential Thrombocythemia (PET-MF) and Polycythemia Vera (PPV-MF). Notably, MPN patients experience increased risk of thrombosis, disease progression (from ET/PV to MF), second neoplasia and evolution to acute leukemia. Although MPN patients overall have reduced life expectancy compared with the general population, the relative survival rate is lower in PMF compared with PV, and in PV compared with ET. Of note, survival of patients with WHO-defined ET is similar to that of the sex- and age-standardized European population. It has been shown that excess mortality in MPN patients is mainly due to death from hematologic malignancies or Figure nfections, and in young patients also from cerebrovascular and cardiovascular diseases [1,2,3,4,5,6]. Sex influences MPN presentation, symptom burden and natural history [7,8]. 

“Driver” mutations in three genes (*Janus kinase* (*JAK)2, calreticulin (CALR)* and *myeloproliferative leukemia virus oncogene* (*MPL*)) that activate the JAK2 signaling pathway have been detected in MPN. However, around 10% of MF and ET patients are unmutated for the *JAK2*, *MPL* and *CALR* genes (“triple negative” patients). The most frequent mutation, *JAK2V617F*, activates the three main myeloid cytokine receptors (erythropoietin receptor, MPL and granulocyte colony-stimulating factor receptor), whereas *CALR* or *MPL* mutants are associated to MPL activation only. For this reason, the *JAK2*V617F mutation is observed PV, ET, and PMF whereas *CALR* and *MPL* mutants are linked to ET and PMF. Additionally, other mutations in genes involved in splicing, epigenetic regulation, and signaling cooperate with the three MPN drivers and play a key role in the MPN pathogenesis and disease progression/leukemic transformation [9,10]. However, independently of the mutation status, a hyper-activation of the JAK-STAT pathway, that transduces most hematopoietic and inflammatory cytokines, is observed in MPN [11]. 

### 1.2. MPN Microenvironment: Inflammation, Immunity and Beyond

The microenvironment plays a key role in MPN pathogenesis. In fact, beyond molecular pathogenesis, MPN are characterized by a state of chronic inflammation due to the continuous release of inflammation factors from in vivo activated leukocytes and platelets/megakaryocytes. Interestingly, this state of chronic inflammation involving the malignant HSC and the non-malignant/malignant microenvironment has been indicated as the main contributor in MPN initiation/clonal evolution. In fact, strong evidence suggests that stromal cells are primed by the malignant hematopoietic clone, which, in turn, conditions the stroma to create a favorable microenvironment that, in turn, nurtures and protects the malignant cells [12,13,14,15]. Many growth factors/cytokines are elevated in MPN patients, including interleukin (IL)-1, IL-6, IL-8, IL-10, IL-11, IL-17, Tumor Necrosis Factor (TNF)-α and transforming growth factor (TGF)-β. Most of the cytokines are either pro-inflammatory or directly pro-fibrotic factors (such as TGF-β). Notably, malignant and non-malignant cell types such as monocytes and megakaryocytes, as well as cells of the bone marrow niche, contribute to the genesis of chronic inflammation, promote disease progression and cause the related morbidity. The overexpression of these pro-inflammatory cytokines is particularly present in MF where increased levels of various pro-inflammatory factors correlate with decreased survival and are the cause of constitutional symptoms; however, there is considerable overlap between the cytokine profile of MF and those of PV and ET. In PV, levels of IL-1β, IL-12 and interferon (IFN-α or γ) have been shown to correlate with hematocrit, leukocytosis and risk of thrombosis [16,17,18,19,20,21,22,23,24,25,26].

Notably, in MPN chronic inflammation is associated with significant immune derangement. Cells of the innate and adaptive immunity, including innate lymphoid cells (ILC), dendritic cells, monocytes, and T/B cells show quantitative and functional abnormalities [27,28]. Specifically, reduced levels of circulating natural killer (NK) cells were observed in MPN patients. These NK cells are also functionally impaired [29,30,31]. As demonstrated by prior studies, in MPN monocytes, regardless of their mutational status, (i) secrete large amounts of cytokines, such as TNF-α, TGF-β and IL-10 [23], (ii) show impaired response to the anti-inflammatory IL-10 [32] and (iii) stimulate osteoclastogenesis [33]. Additionally, in MF the infection-driven response of circulating monocytes is defective since monocytes from *JAK2V617F*-mutated patients show an altered expression of chemokine C-C chemokine receptor type 2 (CCR2), C-X-C motif chemokine receptor 3 (CXCR3), CCR5 and cytokine (TNF-α-R, IL-10-R, IL-1β-R, IL-6-R) receptors. Furthermore, their ability to produce and secrete cytokines (IL-1β, TNF-α, IL-6, IL-10) under lipopolysaccharides stimulation is severely impaired [34]. Interestingly, CD56+CD14+ pro-inflammatory monocytes have recently been identified in ET as a source of increased C-X-C chemokine ligand-1 (CXCL1) levels, which correlate with evolution towards MF [35]. Notably, monocytosis is an independent unfavorable prognostic factor for overall survival in patients with PV and MF [36,37]. In MF Romano et al. demonstrated quantitative and functional abnormalities of circulating lymphocyte subsets, such as T helper (Th)1, Th17, NK and ILC, as well as a reduced ability of monocytes to differentiate into fully committed dendritic cells [38]. In MPN, myeloid-derived suppressor cells were also increased and were associated with specific suppressive activity against autologous T cells [39]. Interestingly, protective “MPN-specific” T cells, selectively targeted against *JAK2V617F* and *CALR* mutated cells, have been recently described. Specifically, spontaneous *CALR* mutant-specific CD4+ T lymphocytes are in the peripheral blood of many MPN patients, and ex vivo co-cultures of T cells specific to *CALR* mutations, as well as *JAK2V617F*-specific CD8+ T cells, with *CALR* or *JAK2* mutated cells induces recognition and elimination of the mutated cells [40,41,42]. Consistently, a prior study demonstrated that CALR mutation-specific CD4+ and CD8+ T cells are in the peripheral blood of patients with CALR mutated MPN; however, their functional impairment may be due to the expression of exhaustion markers (programmed cell death receptor 1 (PD-1) or cytotoxic T-lymphocyte-associated antigen-4 (CTLA-4)) [43]. Moreover, contrasting results have been reported for regulatory T cells. It has been published that the proportion, phenotype and function of circulating CD4+ CD25+ Foxp3+ T cells are in the normal range and are increased after IFN-α treatment in MPN [44]. More recently, CD4+ CD127lowCD25highFOXP3+ regulatory T cells were found to be reduced in MPN patients compared to healthy subjects [45]. Additionally, Romano et al. recently described that circulating regulatory T cells of MF patients were reduced and dysfunctional with increased cytokine production associated with a reduced ability to suppress the proliferation of autologous effector T cells [38]. Altogether, these findings suggest that the immune system is deeply dysregulated in MPN and that tumor immune evasion likely contributes to MPN development and progression. 

Finally, a growing number of studies also demonstrate that the bone marrow niche of MPN is altered at multiple levels, contributing to the survival and maintenance of the mutated HSC [46]. Specifically, functional abnormalities of the vascular and endosteal niches have been described. Mutated HSC can reshape the vascular niche by upregulating several factors promoting angiogenesis and fibrogenesis in order to create a “nurturing” microenvironment. In particular, TGF-β1, mainly produced by megakaryocytes, can induce fibrosis by skewing the activity of mesenchymal stromal cells (MSC) towards fibroblast genesis and by increasing collagen generation and deposition. Platelet-derived growth factor (PDGF) and Vascular Endothelial Growth Factor (VEGF), in turn, stimulate myofibroblast and megakaryocyte development. CXCL4 also plays a key role in the generation of bone marrow fibrosis by upregulating pro-fibrotic pathways in megakaryocytes and by inducing glioma-associated oncogene homolog 1 (Gli1)+ MSC migration and differentiation into myofibroblasts [47]. Additionally, based on the fact that bone marrow nestin(+) MSCs innervated by sympathetic nerve fibers regulate normal HSC, Arranz et al. demonstrated that abrogation of this regulatory circuit is essential for MPN pathogenesis. Sympathetic nerve fibers, supporting Schwann cells and nestin(+) MSC are reduced in the bone marrow of MPN patients and mice expressing the human *JAK2V617F* mutation in HSC. This MSC reduction is due to bone marrow neural damage and Schwann cell death triggered by interleukin-1β produced by mutant HSC [48]. Recently, in the *JAK2V617F*-mutated MPN, it has been described that monocytes can stimulate osteoclastogenesis, promoting the survival and expansion of the malignant cells over normal hematopoiesis [33]. In addition, based on a murine model of MPN, it has been reported an abnormal osteoblast expansion due to overstimulation by MSC, associated with overproduction of inflammatory cytokines, promotion of fibrogenesis and downregulation of CXCL12 expression, leading to the creation of a “self-nurturing” niche [49].

### 1.3. MPN Microenvironment: The Power of Extracellular Vesicles

Cell-to-cell communication plays a key role in tissue homeostasis, but also in several disorders including cancer. The cell-cell interplay mainly relies on cell-cell contact, soluble signals and extracellular vesicles (EVs). EVs are lipid bilayer particles produced by all cell types, usually after activation. EVs function as players in either short- or long-distance intercellular communication [50,51] since EV structure can prevent degradation of bioactive molecules [52]. Indeed, the EV-mediated cross-talk occurs via the trafficking of EV-associated bioactive molecules such as nucleic acids, proteins, metabolites, and lipids [50]. EVs have been detected in various biological fluids including blood, urine and saliva. In blood, the EVs of megakaryocyte and platelet origin are the most abundant [53,54]. However, EVs derived from leukocytes, red blood cells and endothelium are also commonly detected. The International Society of Extracellular Vesicles have classified EVs into three main groups: (1) exosomes, small vesicles with diameters ≤100–150 nm that are formed inside multivesicular bodies; (2) microvesicles, medium size vesicles of plasma membrane origin ranging in diameters up to 1000 nm; and (3) apoptotic bodies, large vesicles with diameters > 1000 nm that are produced by cells undergoing apoptosis [55]. Consistently, based on diameter, biogenesis and cargo, different classes of EVs have been identified, including either Small (S)- or Large (L)-EVs [56,57,58]. Exosomes originate from the endosomal system with a progressive accumulation of intraluminal vesicles within large multivesicular bodies. The multivesicular bodies in turn can fuse with lysosomes or, alternatively, with the cell membrane releasing the intraluminal vesicles into the extracellular space. The generation of multivesicular bodies is driven by Endosomal Sorting Complex Required for Transport (ESCRT)-dependent and -independent pathways [59]. On the other hand, microvesicles generation occurs through the budding of the external cell membrane and the Rho-related coiled helix forming protein kinase (Rho-Rock) pathway seems to play a pivotal role in this process. EV cargo depends on cell type, patho-physiological conditions and drives the functional role of EVs. Overall, the composition of EVs includes proteins involved in the formation of multivesicular bodies’ formation and membrane transport and fusion, tetraspanins, cytoskeletal components and proteins of cytosolic origin. EVs also carry nucleic acids (mRNA, microRNA, long non-coding (lnc)RNA, and double strand (ds)DNA) and selected lipids. Of note, EV lipids play a structural role in forming the EV membranes, but are also precursors of important signaling lipid mediators [60,61,62]. Several mechanisms of EV uptake by target cells have been described including membrane fusion, receptor-ligand interaction and endocytosis [63]. However, the procedures for EV isolation, storage, recovery, and the characterization from biofluids still need to be standardized for clinical applications. Excellent reviews on the bio-molecular and functional characteristics of EVs as well as on the techniques in EV isolation and characterization have been recently published [64,65,66,67,68]. 

Growing interest is focused on EVs and their potential impact on the regulation of normal and tumor microenvironment. Recent findings demonstrated EVs playing critical roles in cancer development (including blood cancers), progression and drug resistance. This is mainly due to their unique mechanism of cell-cell communication and their potential role as disease biomarkers. Specifically, EVs have been suggested to orchestrate the complex interplay between tumor cells and the microenvironment with a pivotal role in “education” and “crafting” of the microenvironment [69,70,71]. Of note, EVs are part of the inflammatory network where adenosine triphosphate (ATP) is among the molecules involved. ATP plays a role in EV biogenesis; in addition, as cargo of EVs, it might facilitate tumorigenesis [72]. Importantly, it has been shown that the genetic and phenotypic intratumor heterogeneity is also reflected in the EVs’ repertoire. Although L-EVs have been less investigated as compared to S-EVs and their role is not completely understood, L-EVs have progressively gained increasing interest in the last years. Indeed, L-EVs have been shown in cancer including breast cancer, prostate cancer, glioblastoma, pancreatic cancer, colon cancer, melanoma and leukemia [56]. However, only large oncosomes (LO) have been described to be released exclusively by cancer cells. LO derive from cell surface membrane blebbing and their size is higher than classical EVs (exosomes (30–150 nm) and microvesicles (100–1000 nm)), ranging from 1 to 10 μm. LO should be more accurate in tumor monitoring than classical L-EVs because they are mainly released by cancer cells. Notably, LO detection can discriminate between healthy and cancer cells/tissues, particularly in the prostate cancer model, and LO shedding is associated with aggressive features in prostate cancer and glioblastoma models [58,73,74,75,76,77,78]. 

Notably, EVs affect both normal and malignant hemopoiesis [79]. Indeed, EVs derived from blood cancer cells or blood cancer’s microenvironment have been shown to functionally regulate key processes including coagulation, angiogenesis, immunity and chemoresistance [69,80]. Recently, selected studies (as described below) have provided relevant information for elucidating the potential role of EVs in MPN. However, the regulatory mechanisms of various EV-associated bioactive molecules and the in vivo pathologic functions of EVs derived from the malignant hemopoietic clone of MPN or its microenvironment are not yet fully understood. Nevertheless, these studies have provided a strong rationale for profiling cargo molecules in MPN as a new tool of liquid biopsy with diagnostic and prognostic potential. Here, we provide a comprehensive overview of EVs in MPN highlighting their functional role as liquid biopsy biomarker and in the “regulation” and “crafting” of both malignant cells and the microenvironment. In addition, the role of EVs in the pathogenesis of thrombotic complications was also discussed. 

## 2. The EV World of PV

PV is characterized by increased red blood cell mass with suppressed erythropoietin production and bone marrow panmyelosis. The clinical phenotype includes increased risk of thrombosis, splenomegaly and systemic symptoms. Additionally, second tumors and infections may occur in a significant fraction of patients. Over time, PV may progress to acute leukemia or secondary MF [81]. In studies that correlated cytokine levels of PV patients IL-2, soluble IL-2R, and IL-6 correlated with MF transformation from both PV and ET [82]. Annual incidence rate is 0.84 per 100,000; however, prevalence is much higher (1/3.300) due to prolonged life expectancy. PV pathogenesis relies on the *JAK2* gene hyperactivation, driven by the *JAK2V617F* (95% of the cases) or exon 12 mutation (3%). In 2% of the cases, no driver mutations can be detected [83]. Along with a decrease of hemoglobin/hematocrit thresholds, 2016 WHO classification upgraded marrow histology as major diagnostic criteria for PV. Venous/arterial thromboses represent the major clinical complications in PV, causing >40% of all deaths, with a cumulative rate of non-fatal thrombosis of 3.8 events per 100 patients/year. Thrombosis may occur prior to or at diagnosis (around 25% of pts) and/or after diagnosis (around 20–25% of pts) and is the result of the hypercoagulable state associated with an increased amount of blood cells and inflammatory signals. Conventional risk assessment relies upon two patient-related features (age > 60 yr and/or the previous thrombosis); the risk of thrombosis is increased 5–10-fold in high-risk (≥1 risk factor) and by 2–4-fold in low-risk patients. Additionally, it has been described that C-reactive protein correlates with thrombosis and *JAK2V617F* variant allele frequency in a combined PV/ET cohort [84]. Prevention of thrombotic complications is the major focus of PV management. In low-risk PV, phlebotomy and low-dose aspirin are recommended, while high-risk patients should also receive cytotoxic therapy, including Hydroxyurea (HU-in all patients), Interferon-alpha (IFN-in younger patients) and Busulfan (BUS-in the elderly). Ruxolitinib (RUX), a *JAK1/2* inhibitor, is approved for PV patients who are resistant or intolerant to HU [81,85,86,87]. 

The role of EVs in PV has been recently addressed. Ahadon M et al. found that PV patients show an increased percentage of plasma EVs of platelet origin [88]. In addition, comparing the proteomic composition of serum EVs from PV patients and healthy controls, Fel A et al. found that 38 proteins were differentially expressed between the two groups. Specifically, 30 proteins were more abundant in PV EVs and 8 were less abundant. These proteins were principally involved in platelet activation, immune/inflammatory response and pro-coagulant and angiogenic pathways. In addition, the presence of higher abundance of erythrocyte, platelet and monocyte membrane markers suggested an elevated count of EVs of erythrocyte, platelet and monocyte origin in PV [89]. 

Regarding the role of EVs in the hypercoagulable state of PV, Duchemin et al. found that in PV and ET patients the “thrombomodulin resistance” is partly determined by circulating EV. This thrombomodulin-resistance might contribute to the hypercoagulable state observed in MPN patients [90]. Additionally, Tan et al. demonstrated that PV patients have increased circulating lactadherin+ and phosphatidylserine+ EVs, mostly originating from erythrocytes, platelets and endothelial cells. These EVs are highly procoagulant and treatment with HU is associated with a decrease in EV release by erythrocytes and platelets [91]. Moreover, comparing ET, PV and MF, a significantly higher procoagulant activity of circulating EVs was found in MPN patients with the highest level in patients with PV as compared with both ET and MF patients. Remarkably, patients with a history of venous thrombosis have higher EV procoagulant activity. Furthermore, the presence of the *JAK2V617F* mutation was associated with an increased procoagulant activity, as well as the higher *JAK2V617F* variant allele frequency [92]. Interestingly, Zhang W et al. found that in MPN, along with the circulating EVs of platelet, erythrocyte and endothelium origin, also the Tissue Factor (TF)-positive EVs were significantly increased. Of note, EV level was increased in the MPN patients with thrombosis compared with patients without thrombosis. In addition, the presence of the *JAK2V617F* mutation was associated with increased levels of EVs of platelet origin [93]. Consistently, Taniguchi et al. described that plasma levels of procoagulant EVs expressing TF were significantly higher in MPN patients (mostly including PV and ET) suffering thrombotic events than in patients without such events, suggesting that TF-positive EVs may be considered a biomarker of thrombosis. Furthermore, among patients who developed thrombosis and irrespective of patients’ blood counts, TF-positive EVs were significantly higher in patients without cytoreductive therapy than in those receiving cytoreductive therapy [94]. Finally, it is known that arterial thrombotic events are the leading cause of death in patients with *JAK2V617F* MPN. However, the high prevalence of myocardial infarction without significant coronary stenosis or atherosclerosis suggests that vascular function is altered in patients with MPN. Interestingly, a mechanism driven by EVs of erythroid origin has been recently published. Specifically, these authors demonstrated that microvesicles derived from erythrocytes are responsible for increased arterial contraction in response to vasoconstrictive agents in *JAK2V617F* MPN, possibly accounting for the arterial events associated with MPN. This effect is due to overexpression of myeloperoxidase in *JAK2V617F* erythrocyte–derived microvesicles, which, in turn, causes increased endothelial oxidative stress and nitric oxyde pathway inhibition [95].

Taken together, these studies provide evidence that circulating EVs are increased in number and show a critical procoagulant signature in PV, suggesting a role of EVs as a biomarker for thrombotic events. Furthermore, due to their cargo of factors with procoagulant potential, circulating EVs are likely to actively contribute to the development and maintenance of the procoagulant state of PV. Further studies are needed to characterize the biomolecular cargo of EV and to address their role in disease progression and leukemic evolution.

## 3. The EV World of ET

ET is characterized by expansion of the megakaryocyte and platelet compartment. The most prevalent mutation is the *JAK2V617F* in around 60% of ET patients. Mutations in *CALR* are associated with 20–25% of ET patients. Mutations in *MPL* concern 5–10% of ET [96,97,98,99]. Around 10% to 15% of ET patients lack the driver mutations and are called “triple-negative” (TN). A recent NGS study demonstrated that TN ET patients may have low-level driver mutations in the JAK2 and MPL genes but show a unique molecular signature [100]. In addition, a previous study demonstrated that 15% of ET patients harbor one or more non-driver mutations in *SRSF2*, *U2AF1*, *SF3B1* or *TP53*; moreover, the presence of mutations in *SRSF2* and *SF3B1*, *SF3B1* and *U2AF1*, or in *TP53* was associated with significantly lower rates of overall, myelofibrosis-free, and leukemia-free survival, respectively [101]. Progression of ET to MF has been shown and circulating levels of growth related oncogene (GRO)-alpha and epidermal growth factor (EGF) have been described as biomarkers of progression [35]. Importantly, arterial and venous thrombosis are the primary cause of morbidity and mortality in ET. The estimated incidence of thrombosis in ET is around 14% at 10 years, with prevalence at diagnosis of 10–35%. Notably, the presence of the *JAK2V617F* mutation increases the risk of vascular events in ET. Microvascular complications are also observed, with symptoms including erythromelalgia, migraine, and paraesthesia. Bleeding may also occur, particularly in cases with extreme thrombocytosis. Bleeding is typically due to the loss of high molecular weight multimers of von Willebrand factor (vWF) through increased proteolysis by ADAMTS13 and/or increased vWF adsorption on the surface of platelets. The risk of thrombosis is based on a history of thrombosis, the presence of the *JAK2V617F* mutation, age > 60 years, and cardiovascular risk factors. Conventional risk stratification for ET identifies high risk (age > 60 years and/or history of thrombosis) or low risk (absence of either high-risk feature) patients. Only high-risk patients undergo cytoreductive therapy [102,103,104,105,106].

Regarding the role of EVs, increased absolute numbers of platelet-derived microparticles have been firstly described in patients with ET [107]. A further study tested the diagnostic ability of various parameters including the EVs of platelet origin to discriminate between ET and reactive thrombocytosis. They found that, even though the absolute number of platelet microparticles was abnormally increased in ET patients, this parameter failed to discriminate between ET and reactive thrombocytosis [108]. The procoagulant role of EVs has been addressed also in ET. Trappenburg et al. found that ET patients have higher numbers of microparticles of platelet and endothelial origin. In addition, microparticles from ET patients were associated with increased thrombin generation and the CD41/CD62e-positive microparticles were elevated only in ET patients with risk factors for thrombosis, suggesting a role for microparticles in the hypercoagulable state of ET patients. Importantly, they also found that there was no significant correlation between platelet counts and the absolute value of microparticles of platelet origin, suggesting the presence of a regulated process of microparticle formation [109]. Consistently, Marchetti M et al. demonstrated that plasma from ET patients displayed increased thrombin generation potential and procoagulant activity compared to controls. This procoagulant activity was due to the presence of circulating microparticles. Of note, the highest values of microparticles were found in patients with the *JAK2V617F* mutation. However, no difference was observed between the thrombosis and no-thrombosis group [110]. Interestingly, in molecularly annotated ET patients at diagnosis, the *JAK2V617F* mutated patients have more circulating EVs and higher levels of EVs with procoagulant activity than the Calreticulin-mutated and triple negative counterparts. This could be partly explained by platelet activation, as assessed by P-selectin expression on EVs of the *JAK2*-mutated patients. In addition, a relation between EV counts and thrombotic risk was also demonstrated. Comparing the microparticle count and the International Prognostic Score of Thrombosis (IPSET)-thrombosis, patients with high thrombotic risk were associated with higher circulating microparticle count. Interestingly, only the red blood cell- and platelet-derived microparticles were increased in the *JAK2V617F*-mutated patients as compared with the Calreticulin-mutated and triple negative patients [111]. Notably, Zhang et al. demonstrated that in ET the absolute values of EVs of platelet, erythrocyte and endothelium origin and the TF+EVs were significantly increased as compared with the normal counterparts [92]. Moreover, Piccin et al. showed that in ET anagrelide treatment normalizes the proportion of circulating EV of platelet origin [112]. Contrasting results have been published when the proportion of circulating platelet-derived microparticles was correlated with the platelet number. In detail, Trappenburg et al. found that there was no significant correlation between platelet counts and the absolute value of microparticles of platelet origin, suggesting the presence of a regulated process of microparticle formation [109]. Conversely, Connor DE et al. found that the increased platelet-derived microparticle proportions are related to the high platelet count [107]. Whether this is due to the differences in the method of identification of the platelet-derived microparticles remains a matter of discussion. Interestingly, when the nucleic acid cargo of EVs from ET patients was analyzed, it has been found that the profile of circular RNA (circRNA-noncoding RNA molecules that modulate the expression of target genes by sponging microRNAs or directly binding with other RNA-associating proteins, [113]) was altered in bone marrow-derived exosomes of ET patients. Specifically, circDAP3, circASXL1, and circRUNX1 were significantly downregulated in exosomes of ET patients. Of note, circDAP3 is able to inhibit the megakaryocytic differentiation of K562 cells. Importantly, circRNA-encoding genes and miRNA-mRNA networks targeted by these three circRNA were involved in various key biological processes and signaling pathways such as cell proliferation and apoptosis [114]. 

In conclusion, these data demonstrate that circulating EVs of ET patients are abnormally increased, may be considered a biomarker of thrombosis and play a pathogenetic role in the generation/maintenance of the hypercoagulable state of ET patients.

## 4. The EV World of MF

MF is characterized by abnormal proliferation of megakaryocytes (with the aberrant immature feature) and granulocytes. MF patients show, along with severe anemia and cytopenia, bone marrow fibrosis, splenomegaly, extramedullary hemopoiesis and chronic inflammation. Typical clinical manifestations include debilitating systemic symptoms, progressive splenomegaly and transfusion-dependent cytopenia. MF is the most aggressive of the MPN, with severely reduced overall survival with an incidence of 0.58 new cases per 100,000 people/year, and predominantly affects the elderly, with over 60% of diagnosis occurring in >65 year age [115,116]. 

The molecular pathogenesis of MF relates to mutations in three “driver” genes (namely: *JAK2, CALR, MPL*) that cause hyper-activation in the JAK-STAT pathway. In addition, subclonal mutations in five genes (*IDH1/2*, *ASXL1*, *SRSF2*, *EZH2*) are associated with worse outcomes. The absence of the three “driver” mutations is found in around 10% of patients (“triple negativity”). TN MF is associated with an aggressive clinical behavior characterized by a higher risk of developing anemia and thrombocytopenia, poorer outcomes in comparison with patients affected by the other MF molecular subtypes and a high rate of leukemic transformation. The molecular basis of TN remains mostly unknown, although a high molecular complexity has been previously described and rare, alternative, somatic mutations in both *JAK2 exon 14* and *MPL exon 10* have been previously described [117,118,119]. Beyond the molecular pathogenesis, chronic inflammation with abnormal release of pro-inflammatory cytokines has been indicated as main contributor in MF initiation/clonal evolution. Along with International Prognostic Scoring System (IPSS) and DIPSS, GIPSS (genetically-inspired prognostic scoring system) and MIPSS70+ version 2.0 (MIPSSv2; mutation- and karyotype-enhanced international prognostic scoring system) have recently been introduced: GIPSS is based exclusively on mutations and karyotype and identifies four categories. MIPSSv2 includes, in addition, clinical risk factors and identifies five categories [117]. Standard therapy has little effect on the natural history of the disease; MF is still a treatment-orphan disease that may be cured only by allogeneic stem cell transplant in younger selected patients. 

Patients with splenomegaly and systemic symptoms are eligible for therapy with ruxolitinib, a JAK1/2 inhibitor that suppresses clonal myeloproliferation and release of proinflammatory cytokines, reducing splenomegaly and constitutional symptoms in around 50% of patients, without inducing significant reductions in mutation load/marrow fibrosis. However, responses are durable in only 50% of the cases, and therapy may be burdened by significant toxicity [117,120].

Regarding the role of EV in MF, Caivano et al. [121] firstly demonstrated that circulating EVs may represent a novel diagnostic and prognostic biomarker since high serum levels of EVs are detected in the peripheral blood of patients with various types of blood cancers. They found serum EVs to be elevated in acute myeloid leukemia, multiple myeloma, Hodgkin lymphoma and Waldenstrom Macroglobulinemia; serum EVs levels were also slightly increased in MF but their size was lower than those of the normal counterparts. In addition, Zhang W et al. recently demonstrated that circulating microparticles of erythrocyte, platelet and endothelial origin were increased in MF patients as compared with those of healthy donors and patients with PV. In addition, TF+ EVs were also increased [93]. Furthermore, we recently demonstrated that irrespective of mutation status, an increased and decreased proportion of circulating platelet-derived EVs and megakaryocyte-derived EVs is observed in patients with MF. Of note, according to the IPSS risk category, the proportion of megakaryocyte- and platelet-derived EVs of high-risk patients was significantly reduced and increased, respectively, in comparison with the low-risk counterparts. Additionally, the JAK1/2 inhibitor ruxolitinib normalizes the profile of plasma EVs in the MF spleen-responder patients only by increasing the megakaryocyte EVs and decreasing the platelet EV proportions. Importantly, a cut-off value of 19·95% of megakaryocyte-derived EVs discriminates between spleen responders and non-responders, demonstrating that circulating EVs of megakaryocyte origin, as a liquid biopsy assay, may be a potential tool to predict response to ruxolitinib therapy in MF [122]. 

Furthermore, we also demonstrated that distinct EV-driven signals from the microenvironment are capable to promote the TN malignant hemopoiesis. Specifically, we found that in TN patients the in vitro hemopoietic survival, phenotype, and hemopoietic function of circulating CD34+ cells are significantly altered and the CD34+ cells are unresponsive to the inflammatory microenvironment. Of note, the plasma levels of crucial inflammatory cytokines are mostly within the normal range in TN patients. Compared to *JAK2V617F*-mutated patients, the gene expression profiling of the TN CD34+ cells revealed distinct signatures in key pathways such as survival, cell adhesion, and inflammation. In addition, it has been observed the presence of mitochondrial components within plasma EVs and a distinct phenotype in TN-derived EVs compared to the *JAK2V617F*-mutated MF patients and HD counterparts. Notably, only TN EVs promoted the survival of TN CD34+ cells. Along with a specific microRNA signature, the circulating EVs from TN patients were enriched with miR-361-5p, with a role in apoptosis and inflammation pathways [123].

Keeping a focus on signals from the microenvironment, we further found that the ability of monocytes from *JAK2V617F*-mutated MF patients to produce and secrete free and EV-linked cytokines (IL-1β, TNF-α, IL-6, IL-10) under lipopolysaccharides (LPS) stimulation is severely impaired. Interestingly, monocytes from the ruxolitinib-treated patients show normal level of chemokine, IL-10, IL-1β, and IL-6 receptors together with a restored ability to produce intracellular and to secrete EV-linked cytokines after LPS stimulation. Conversely, ruxolitinib therapy does not normalize the LPS-driven secretion of free inflammatory cytokines. Accordingly, upon LPS stimulation, in vitro ruxolitinib treatment of monocytes from MF patients increases their secretion of EV-linked inflammatory cytokines but inhibits the secretion of free inflammatory cytokines. Therefore, in MF the infection-driven response of circulating monocytes is defective. Importantly, ruxolitinib promotes infection-driven cytokine production of monocytes, suggesting that infections following ruxolitinb therapy may not be due to monocyte failure [34]. 

Overall, these limited yet interesting studies support the role of EVs as biomarkers with diagnostic and prognostic significance and highlight the importance of the EV-mediated cross-talk in the microenvironment of MF. Further studies are warranted to decipher the underlying mechanisms and to address the potential clinical importance of EVs in MF.

## 5. Conclusions

**Figure 1:**
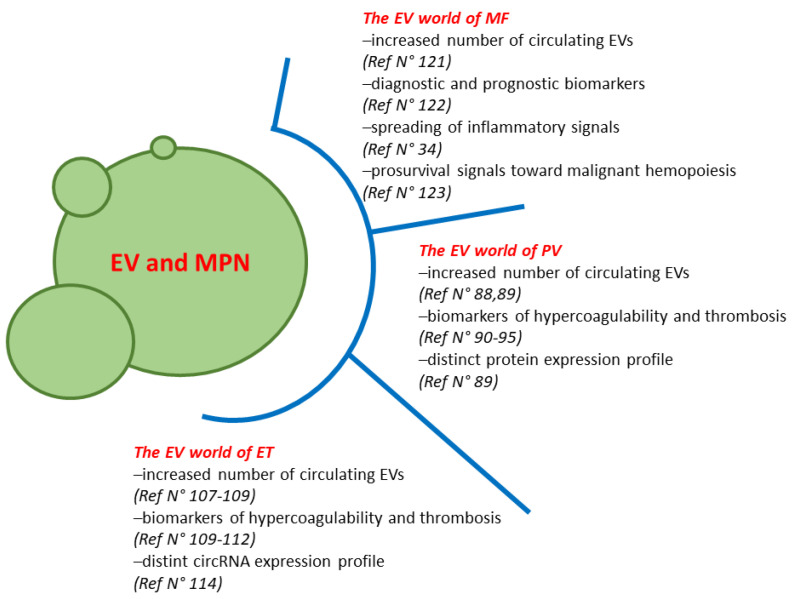
The role of extracellular vesicles in MPN.

Our understanding of the biological complexity of MPN and how this relates to outcome is increasingly improving. Circulating EVs show promise as crucial players in liquid biopsy of MPN. This means that the identification of biomarkers within EV for clinical studies could be crucial in the diagnosis and prognosis of MPN. Overall, most of the published studies on the role of EV in PV and ET are mainly focused on their procoagulant potential. In fact, increasing evidence highlights the contribution of EVs to the pathogenesis of thrombotic complications in MPN patients. This is likely due to the pivotal and well-known role played by EVs in the regulation and “crafting” of vascular reactivity, angiogenesis, inflammation and thrombosis. In MF the circulating EVs may have diagnostic and predictive roles in response to ruxolitinib. In addition, they can functionally promote the survival of the malignant hemopoiesis (Figure 1). However, although there is increasing evidence for the role of circulating EVs in MPN, many questions remain. For example, it is yet to be determined how and whether EVs affects MPN disease progression and myelofibrosis or how EV regulation of hypercoagulability differs among the diseases. Further research is also warranted to clarify the complex relationship between the HSC and cells of the immune/inflammatory microenvironment and the role of the EVs from and to cells of the (bone marrow) microenvironment. In addition, the cargo of proteins, lipids and metabolites/proteins of EVs is still to be deeply investigated in MPN. Answers to the above-described questions would largely improve the use of EVs as diagnostic/prognostic tools and promote the conversion of EV studies into the clinic of MPN. In addition, due to their inherent homing ability and low immunogenicity, EVs may represent attractive and promising candidates to optimize drug delivery in MPN.

## Data Availability

Not applicable.
